# Identifying emerging mental illness utilizing search engine activity: A feasibility study

**DOI:** 10.1371/journal.pone.0240820

**Published:** 2020-10-16

**Authors:** Michael L. Birnbaum, Hongyi Wen, Anna Van Meter, Sindhu K. Ernala, Asra F. Rizvi, Elizabeth Arenare, Deborah Estrin, Munmun De Choudhury, John M. Kane

**Affiliations:** 1 The Zucker Hillside Hospital, Northwell Health, Glen Oaks, NY, United States of America; 2 The Feinstein Institute for Medical Research, Manhasset, NY, United States of America; 3 The Donald and Barbara Zucker School of Medicine at Hofstra/Northwell, Hempstead, NY, United States of America; 4 Cornell Tech, Cornell University, New York, NY, United States of America; 5 Georgia Institute of Technology, Atlanta, GA, United States of America; Department of Psychiatry and Neuropsychology, Maastricht University Medical Center, NETHERLANDS

## Abstract

Mental illness often emerges during the formative years of adolescence and young adult development and interferes with the establishment of healthy educational, vocational, and social foundations. Despite the severity of symptoms and decline in functioning, the time between illness onset and receiving appropriate care can be lengthy. A method by which to objectively identify early signs of emerging psychiatric symptoms could improve early intervention strategies. We analyzed a total of 405,523 search queries from 105 individuals with schizophrenia spectrum disorders (SSD, N = 36), non-psychotic mood disorders (MD, N = 38) and healthy volunteers (HV, N = 31) utilizing one year’s worth of data prior to the first psychiatric hospitalization. Across 52 weeks, we found significant differences in the timing (*p*<0.05) and frequency (*p*<0.001) of searches between individuals with SSD and MD compared to HV up to a year in advance of the first psychiatric hospitalization. We additionally identified significant linguistic differences in search content among the three groups including use of words related to sadness and perception, use of first and second person pronouns, and use of punctuation (all *p*<0.05). In the weeks before hospitalization, both participants with SSD and MD displayed significant shifts in search timing (*p*<0.05), and participants with SSD displayed significant shifts in search content (*p*<0.05). Our findings demonstrate promise for utilizing personal patterns of online search activity to inform clinical care.

## Introduction

The consequences of untreated psychiatric illness can be devastating [1–3]. Behavioral health disorders often present during the formative years of adolescent and young adult development and interfere with the establishment of social, educational, and vocational foundations [4]. While early intervention services have demonstrated the potential to improve outcomes, symptoms often remain unrecognized and untreated for years before receiving effective care [5–8]. Novel screening strategies, supported by technological innovation, are critical to achieving the goal of early identification and treatment.

The emergence of serious mental illnesses, such as schizophrenia and bipolar disorder, are often preceded by periods of anxiety, mood lability, sleep pattern irregularity, trouble concentrating, social isolation, strained interactions with others, and attenuated/subthreshold psychotic and manic experiences [9, 10]. Despite the decline in functioning and established deleterious impact of untreated symptoms, an effective method by which to screen and educate vulnerable individuals has not been established [11, 12]. Clinical interview, assessment scales, patient self-report, and family observation remain the primary sources for assessing early warning signs and are limited by reliance on direct and timely contact with trained professionals, as well as accurate and insightful patient and family recall. These standard approaches to clinical assessment do not allow for objective monitoring of psychiatric symptom emergence and typically do not occur with enough frequency, or at the necessary level of detail, to detect subtle, sub-clinical, and burgeoning symptoms. Early, precise, and noninvasive identification of psychiatric symptom emergence could facilitate the initiation of personalized and proactive intervention strategies.

At the same time, Google Search has emerged as one of the world’s most popular websites, supporting over 660 million daily visitors, and managing over three billion searches daily [13]. Searching online has become the primary resource for youth seeking out mental health related information [14]. This is especially true for stigmatized illnesses such as schizophrenia, as the Internet provides a comfortable and anonymous setting to gather information about symptoms and treatment options [15]. Previous reports have demonstrated that adolescents and young adults with emerging symptoms of psychiatric disorders utilize the Internet first, and most frequently, to gather information prior to receiving psychiatric care, and that they are more likely to search online for information than to discuss their experiences with peers, family, physicians, and mental health clinicians [16–18]. Performing an Internet search may therefore represent one of the first proactive steps towards treatment initiation and could provide a valuable opportunity to impact help-seeking behavior.

Prior work in machine learning has highlighted opportunities to utilize large scale anonymized online search activity to detect content and patterns associated with the emergence and progression of medical illnesses including lung cancer, pancreatic cancer, and Parkinson’s disease [19–21]. These initiatives aim to inform the development of a new generation of digital tools designed to assist in the screening and early identification of individuals developing medical health conditions. Similar computational methods have identified associations between social media activity and behavioral health [22–29]. Few studies to date, however, have explored the link between search activity and psychiatric illness, beyond retrospective self-report [30]. Furthermore, while promising, internet activity research to date has been limited by the fact that it has been conducted nearly exclusively using search data from anonymous individuals who self-report a diagnosis online, and has yet to be carried out in real world clinical settings, using participant-contributed search data, with clinically validated symptoms and diagnoses.

This study aimed to explore the feasibility of utilizing online search archives as a tool to identify emerging psychiatric symptoms. This knowledge would support the development of resources designed to inform screening procedures for individuals with emerging mental illness earlier along their trajectory to care. We hypothesized that significant differences in the *content*, *timing*, *and frequency* of online activity would differentiate participants with schizophrenia spectrum disorders (SSD) from those with mood disorders (MD) and healthy volunteers (HV). Additionally, we hypothesized that significant changes in the content and behavioral patterns of search activity would exist within individuals with SSD and MD in the period of time closest to their first hospitalization consistent with escalating psychiatric symptoms during that time period.

## Materials and methods

Participants between the ages of 15 and 35 years, diagnosed with a schizophrenia spectrum disorder or a non-psychotic mood disorder, were recruited from The Zucker Hillside Hospital/Northwell Health inpatient and outpatient psychiatric departments. Most participants with SSD were recruited from the Early Treatment Program (ETP), Zucker Hillside’s specialized early psychosis intervention clinic. Additional participants (3) were recruited from a collaborating psychiatric clinic located in East Lansing, Michigan. Healthy volunteers who had already been screened for prior studies were recruited. Additional HV’s (n = 10) were recruited from the University of North Carolina. Recruitment occurred between March 2016 and December 2018. Written informed consent was obtained for adult participants and legal guardians of participants under 18 years of age. Assent was obtained for participating minors. Participants were fully informed of the potential risks, benefits, and alternative options available, as well as strategies to mitigate risks. Decisional capacity to consent was determined through clinical assessment, as well as via completion of a short quiz, designed to assess one’s understanding of research procedures, conducted prior to consenting to participate. The study was approved by the Institutional Review Board (IRB) of Northwell Health (the coordinating institution) as well as local IRBs at participating sites.

Participation involved a single study visit. Participants were asked to export their search archive by logging on to their Google account to request their search history. Archives include all historical search activity including the content and timing of search queries. Diagnoses and dates for the first psychiatric hospitalization were obtained through participants’ medical records.

Given the goal of identifying changes in search activity associated with escalating psychiatric symptoms, 52 weeks’ worth of search data prior to the first psychiatric hospitalization was extracted from each participant, operating under the expectation that at some point during that year, psychiatric signs and symptoms emerged and progressed to the point of necessitating inpatient intervention. One year was selected as it represents a period of time long enough to establish a baseline level of search activity, and to identify changes in the weeks closest to hospitalization. For HV (who were never hospitalized), the midpoint of the first hospitalization dates across all patients (N = 74) was utilized to mitigate the potential temporal effects on search patterns, such as functional changes in the search platform and search data logging systems over time. This resulted in using November 9, 2015 as the anchor date for healthy participants in our dataset.

Our analysis consisted of (1) between-group comparisons among SSD, MD, and HV to examine group-level differences, and (2) within group comparisons by comparing a period of “relative health” (6 month furthest away from hospitalization) to periods of “relative illness”, closest to the date of the first psychiatric hospitalization.

Both sets of comparisons were conducted on the frequency, timing, and content of searches. We extracted search frequency and timing distributions from the meta data (i.e. timestamps). For search content, we used Linguistic Inquiry and Word Count (LIWC), a well validated language analytic tool, which extracts 93 variables pertaining to word usage, known to be associated with emotion, mood, and behavior [31, 32]. Given the number of comparisons tested, we implemented the two-stage Benjamini and Hochberg [33] procedure to control the false discovery rate (FDR). Specifically, we used the implementation from the statsmodels Python library [34] and set the family-wise error rate to be 0.05.

### Data preprocessing

A total of 132 (44 SSD, 41 MD, 47 HV) search archives were available for analysis. Participants with 30 or more weeks of zero search activity during the 52-week period were excluded (n = 27). The final dataset consisted of 405,523 searches across 105 participants. Demographic information of included participants is shown in Table 1.

**Table 1:** Participant demographics.

	SSD	MD	HV	Full Sample
**N**	36	38	31	105
	**Mean (SD)**			
**Age**	23.11 (3.3)	19.48 (3.1)	25.72 (4.8)	23.12 (4.2)
**Years of Education**	13.58 (1.8)	13.34 (2.1)	16.41 (1.9)	14.29(2.3)
	**n (%)**			
**Male**	22 (61)	10 (26)	11 (35)	43 (41)
**Race/Ethnicity**				
African American/Black	16 (44)	7 (18.4)	5 (16.1)	28 (27)
Asian	5 (13.9)	5 (13.2)	6 (19.4)	16 (15)
Caucasian	12 (33.3)	11 (28.9)	18 (58.1)	47 (45)
Mixed race/Other	3 (8.3)	9 (23.7)	2 (6.4)	14 (13)
Hispanic	9 (25)	11 (28.9)	0 (0)	20 (19)
**Diagnosis**				
Schizophrenia	16 (15)	0 (0)	0 (0)	16 (15)
Schizophreniform	8 (8)	0 (0)	0 (0)	8 (8)
Schizoaffective	1 (1)	0 (0)	0 (0)	1 (0)
Brief Psychotic Disorder	2 (2)	0 (0)	0 (0)	2 (2)
Unspecified SSD	9 (9)	0 (0)	0 (0)	9 (9)
Bipolar I Disorder	0 (0)	5 (5)	0 (0)	5 (5)
Major Depressive Disorder	0 (0)	33 (32)	0 (0)	33 (31)

## Results

### Between-group differences in search (frequency, timing, and content)

#### Frequency of search activity

**Fig 1:**
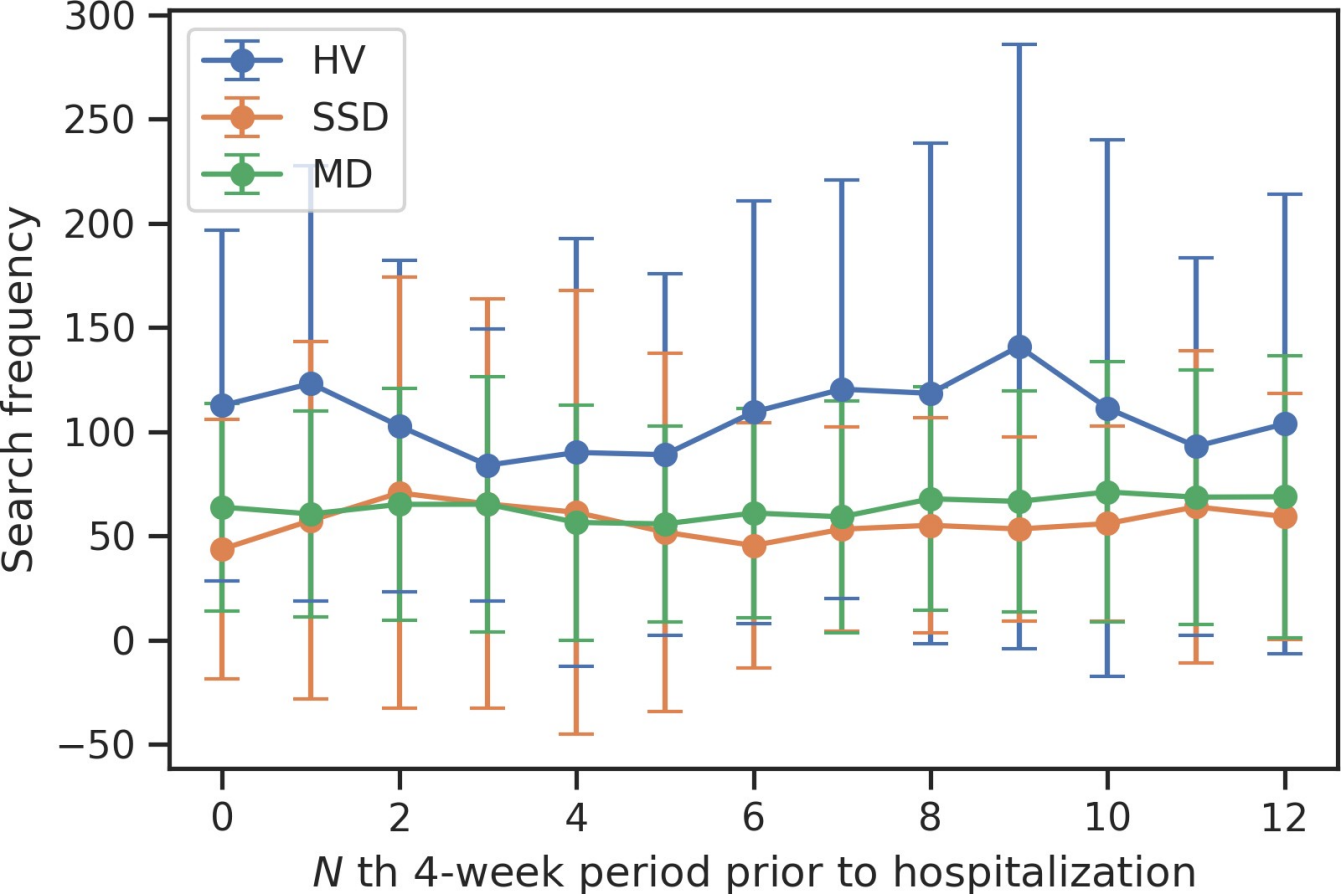
Search frequency across groups over 52 weeks.

Across 52-weeks (Fig 1), HV showed significantly higher search frequency on average compared to both SSD (Post-hoc Tukey: T = 19.51, *p* = 0.001) and MD (Post-hoc Tukey: T = 16.76, *p* = 0.001). HV also showed significantly higher variability of search frequency compared to MD (T = 2.83, *p* = 0.006) across the 52-weeks (averaged standard deviations across weeks: HV = 50.74, MD = 30.12, SSD = 36.36). Education, sex, and age were not associated with search frequency among MD and HV participants. Among SSD participants, those who completed high school (n = 25) searched more often than those who did not (n = 11), and relatedly, young adults with SSD, 20 years and older (n = 28), searched more than adolescents (n = 8).

#### Timing of search activity

**Fig 2:**
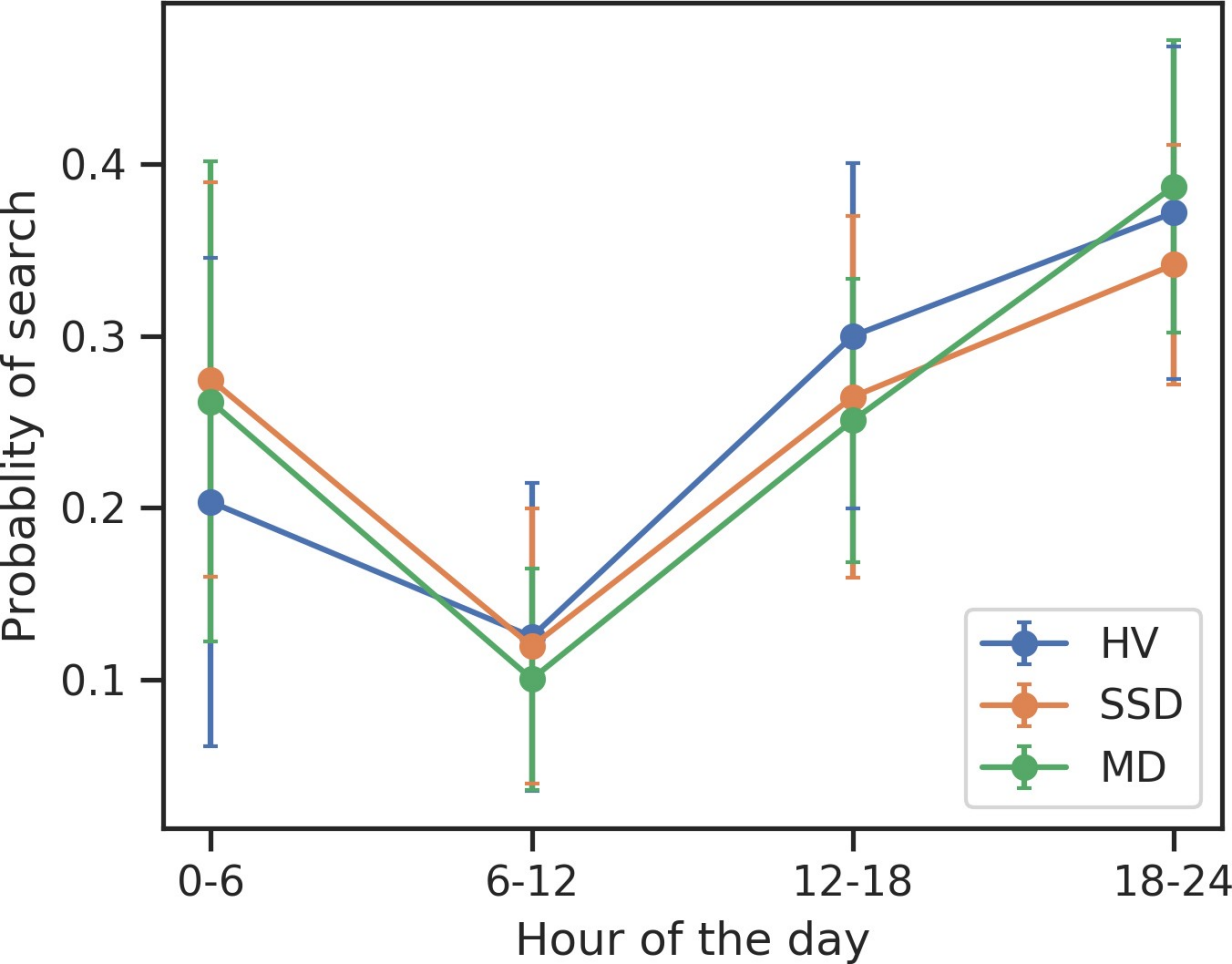
Search timing across groups over 52 weeks.

Over 52 weeks (Fig 2), we found that SSD participants search significantly more during the 12am-6am period (T = 2.24, *p* = 0.029) compared to HV. Additionally, MD participants searched significantly less than HV from 12pm-6pm (T = -2.20, *p* = 0.03) and significantly more than SSD from 6pm-12am (T = 2.48, *p* = 0.015). Education, sex, and age were not associated with search timing among MD, SSD, and HV participants.

#### Content of search activity

Over 52-weeks (Table 2), we identified several linguistic differences in search content across groups. Participants with SSD were significantly more likely to search using words related to perception (T = 3.08, *p = 0*.*025)* and use first (T = 3.01, *p* = 0.03) and second person pronouns (T = 3.45, *p* = 0.011) compared to HV. Participants with MD were significantly more likely to search using words related to negative emotions (T = 2.94, *p* = 0.028), sadness (T = 3.01, *p* = 0.026), and death (T = 2.71, *p* = 0.046), and use first (T = 3.41, *p* = 0.010) and second (T = 3.22, *p* = 0.016) person pronouns compared to HV. HV were significantly more likely to search using more words compared to SSD (T = 3.57, *p* = 0.009) and MD (T = 3.18, *p* = 0.018), use more punctuation compared to SSD (T = 3.53, *p* = 0.009) and MD (T = 3.27, *p* = 0.015), and use common online abbreviations (i.e., b/c for “because”) compared to SSD (T = 3.42, *p* = 0.011).

**Table 2:** Linguistic differences in search content across 52 weeks.

HV > MD	HV > SSD	MD > HV	MD > SSD	SSD > MD	SSD > HV
Word count (*p* = 0.018) Analytic (*p* = 0.006) Words per sentence (*p* = 0.028) Punctuation (*p* = 0.015) Period (*p* = 0.018) Dash (*p* = 0.023)	Word count (*p* = 0.009) Analytic (*p* = 0.009) Words per sentence (*p* = 0.013) We (*p* = 0.023) Informal (*p* = 0.013) Net speak (*p* = 0.011) Punctuation (*p* = 0.009) Period (*p* = 0.009) Dash (*p* = 0.021)	Authentic (*p* = 0.010) Function (*p* = 0.006) Total pronoun (*p* = 0.006) Personal pronouns (*p* = 0.006) I (*p* = 0.010) You (*p* = 0.016) Prep (*p* = 0.040) Aux verb (*p* = 0.006) Adverb (*p* = 0.006) Negate (*p* = 0.006) Verb (*p* = 0.006) Interrogation (*p* = 0.027) Quant (*p* = 0.045) Neg emotion (*p* = 0.028) Sad (*p* = 0.026) Cog process (*p* = 0.027) Cause (*p* = 0.046) Focus present (*p* = 0.006) Death (*p* = 0.046)	Filler (*p* = 0.045)	Semi-Colon (*p* = 0.045)	Authentic (*p* = 0.009) Total pronoun (*p* = 0.009) You (*p* = 0.011) I (*p* = 0.030) Perception (*p* = 0.025) Focus Present (*p* = 0.009)

### Within-group differences in search (frequency, timing, and content)

To explore within group differences in search frequency, timing, and content, search data was aggregated and averaged over one-week intervals. The periods of time within 6 months (24 weeks) closest to hospitalization were defined as periods of “relative illness” as we would expect symptoms to be most prominent during this time, culminating in hospitalization. These periods were compared to periods of “relative health,” which consisted of data from the six months (25–52 weeks) furthest away from hospitalization.

#### Frequency of search activity

No significant differences in search frequency were found between periods of relative illness and periods of relative health in all three groups using repeated measures ANOVA and paired t-tests.

#### Timing of search activity

**Fig 3:**
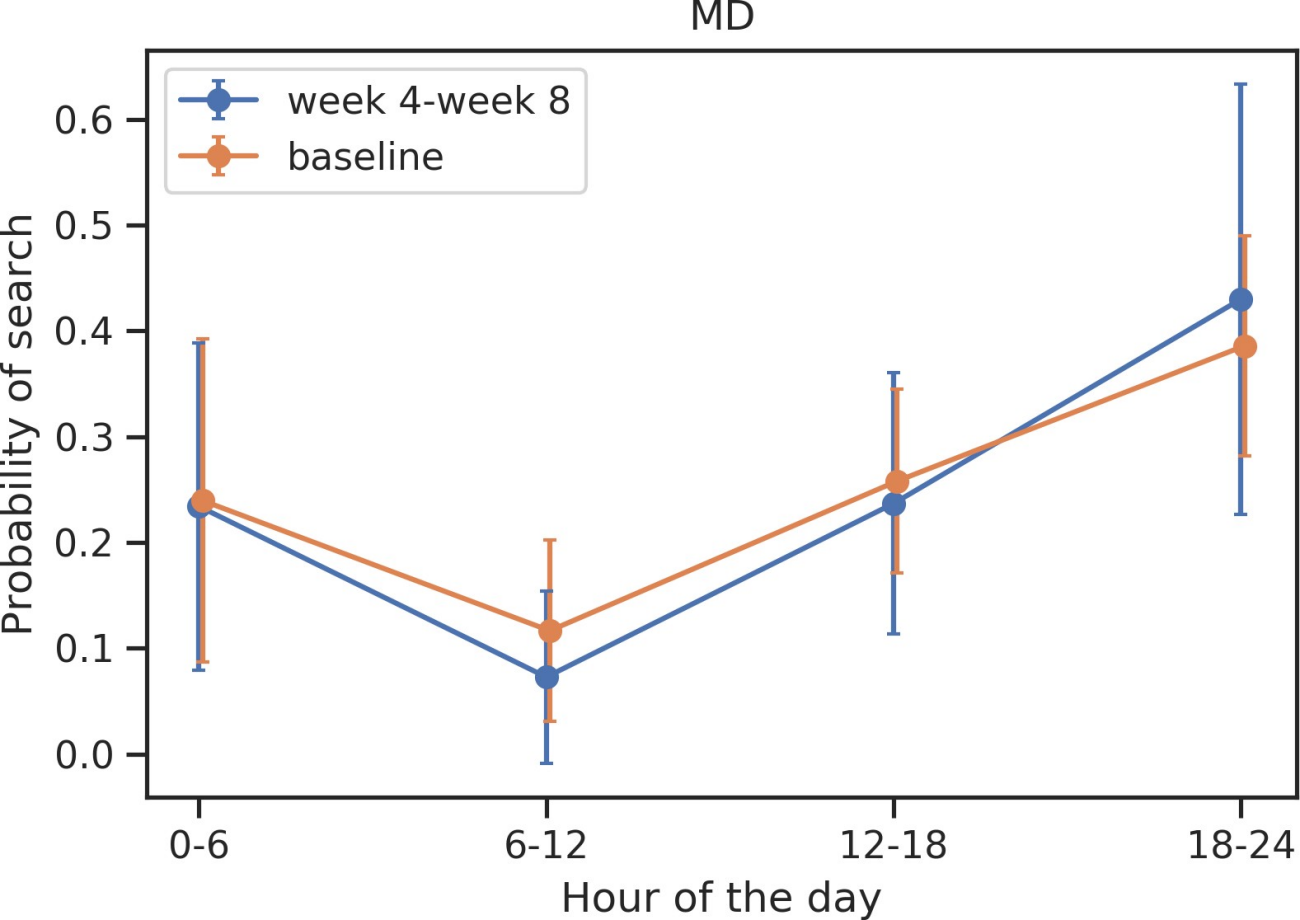
Shifts in timing of search activity across 24 hours in participants with MD.

**Fig 4:**
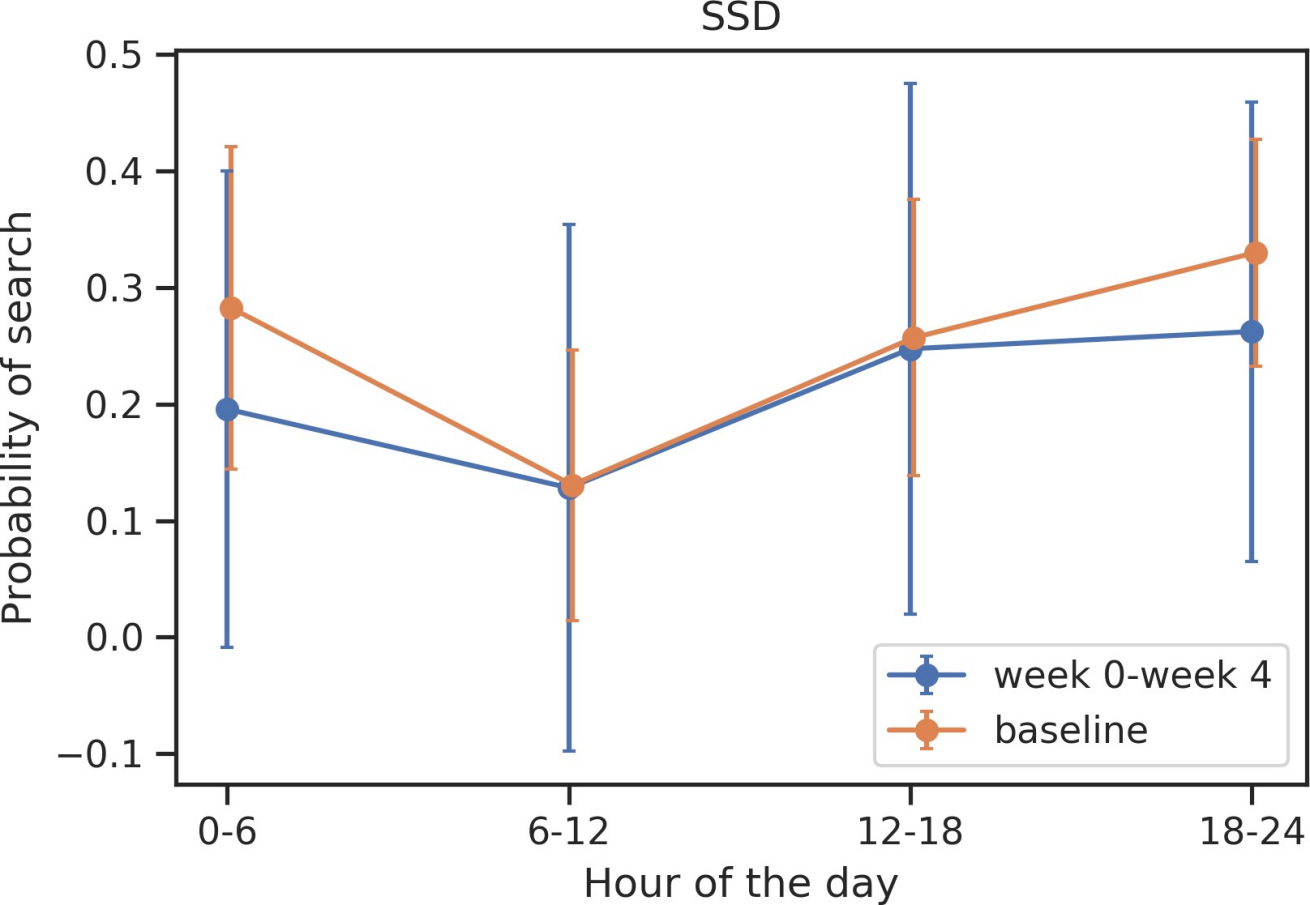
Shifts in timing of search activity across 24 hours in participants with SSD.

Significant shifts were identified in the timing of search activity in participants with MD and SSD closer to hospitalization (Figs 3 and 4). Compared to periods of relative health, participants with MD searched significantly less (T = -3.19, *p* = 0.003) during the morning hours (6am-12pm) during periods of relative illness. Compared to periods of relative health, participants with SSD search significantly less (T = -2.30, *p* = 0.03) during the early morning hours (12am-6am) during periods of relative illness.

#### Content of search activity

**Fig 5:**
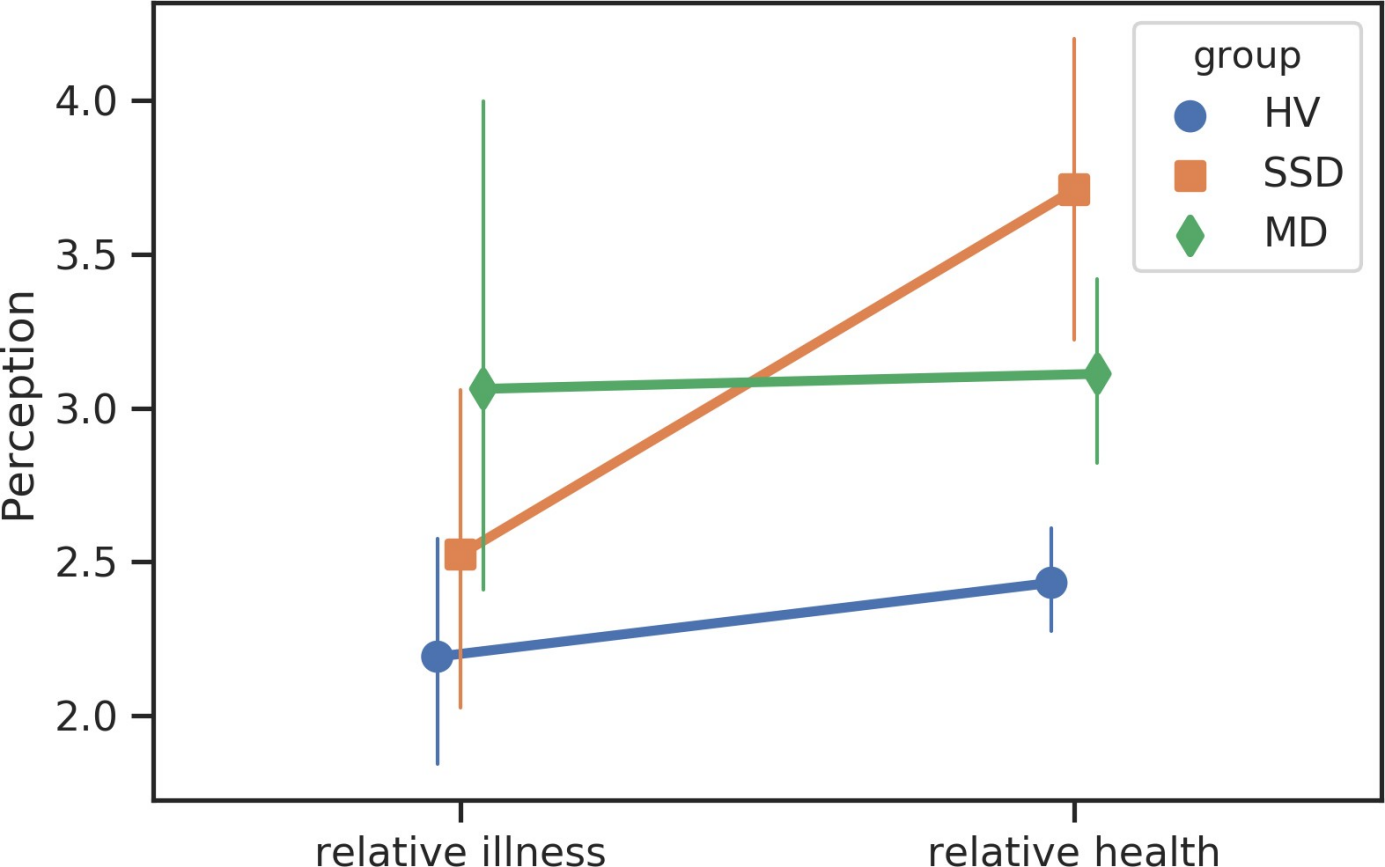
Changes in search content corresponding to “perception”.

**Fig 6:**
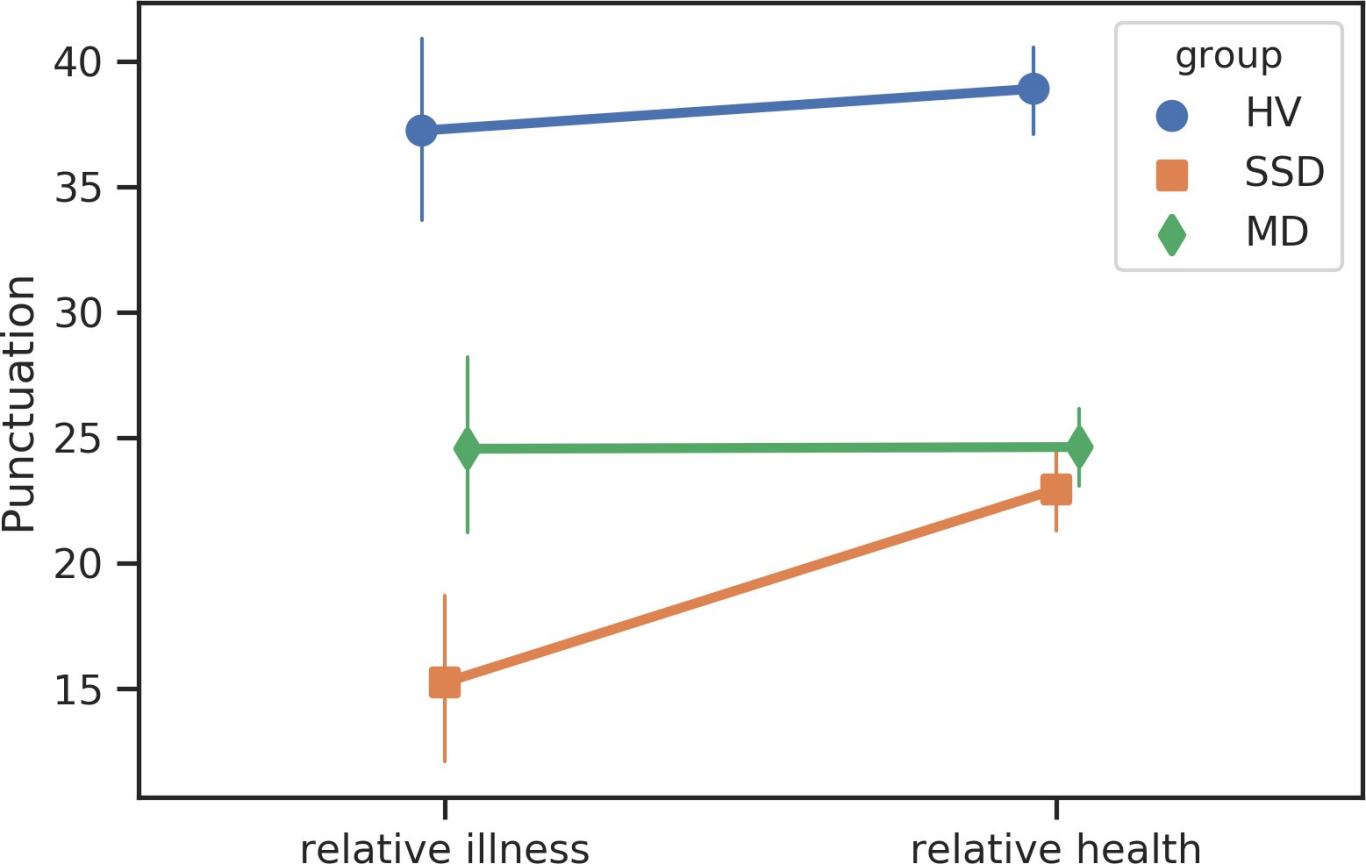
Changes in search content corresponding to “punctuation”.

**Fig 7:**
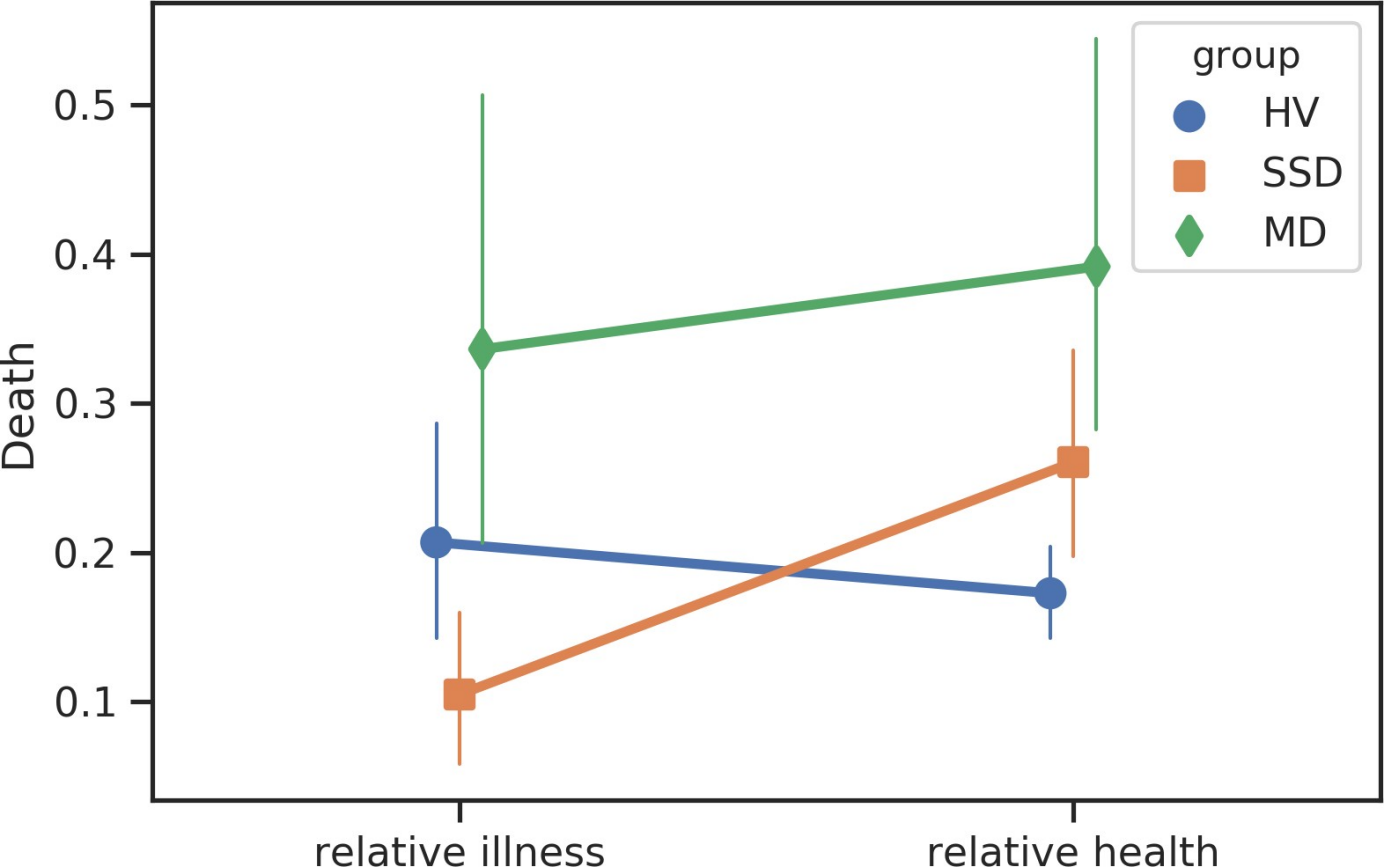
Changes in search content corresponding to “death”.

We identified several significant linguistic shifts in search content among participants with SSD prior to the first psychiatric hospitalization (Table 3). Participants with SSD were less likely to use punctuation (T = -4.13, *p* = 0.0025), less likely to search for terms related to “seeing” (T = -3.79, *p* = 0.01), “anger” (T = -3.47, *p* = 0.023), “negative emotions” (T = -3.15, *p* = 0.04), “perception” (T = -3.30, *p* = 0.03), and “death (T = -3.54, *p* = 0.04),” in the 12 weeks prior to hospitalization compared to periods of relative health (Figs 5–7). No significant shifts in search content were identified among the MD participants.

**Table 3:** Within group changes in content for SSD (comparing periods of relative illness to periods of relative health).

	Weeks prior to hospitalization		
	Week 0–4	Week 4–8	Week 8–12
Content of searches (Relative health > Relative illness)	Quote (*p* = 0.025) Comma (*p* = 0.000008) Punctuation (*p* = 0.0025)	Neg emotion (p = 0.04) Anger (p = 0.025) Perception (p = 0.03) See (p = 0.01)	Death (p = 0.04)

## Discussion

In this study, we explored the potential for online search activity to serve as a tool to identify emerging behavioral health disorders. Our results suggest significant differences exist in the timing, frequency, and content of search activity a year in advance of the first psychiatric hospitalization for participants with SSD and MD, when compared to HV. Furthermore, in the weeks closest to the date of the first hospitalization, significant shifts in language occurred in participants with SSD, and significant shifts in timing occurred in individuals with SSD and MD. While Google data alone is not meant diagnose psychiatric conditions, results demonstrate the potential for online search activity to be used in conjunction with clinical information to inform clinical decision making. Similar to the way a physician might use an x-ray or blood test to inform health status, search data may one day serve as a viable screening tool to better gauge risk factors associated with the later development of psychiatric conditions. Identifying emerging psychiatric symptoms early, before they have an opportunity to escalate to the point of necessitating hospitalization, is our best chance at transforming trajectories to care and improving behavioral healthcare experiences and outcomes for patients.

Individuals with SSD and MD demonstrated significantly fewer searches in the year leading up to the first psychiatric hospitalization as compared to HV, who searched over twice as much. Counter to our hypothesis, no significant changes in search frequency were noted in participants with SSD and MD as psychiatric symptoms escalated necessitating a psychiatric hospitalization. Decreased search activity may be related to very early budding psychiatric symptoms including reduced motivation, increased fatigue, or decreased interest and engagement with one’s environment [35–37]. Additionally, individuals with SSD are known to experience cognitive deficits early in the course of illness development, which may contribute to reduced search activity [38, 39]. These subtle changes may occur well in advance of the first psychiatric hospitalization. In contrast, reduced search activity may represent a longstanding risk factor contributing to the later emergence of a psychiatric disorder. To address these questions, future research will need to extract search data several years in advance of the first psychiatric hospitalization, and to prospectively collect symptom rating scales in individuals earlier along the course of illness development.

Compared to HV, participants with SSD and MD searched at different times throughout the day. Temporal differences date back at least a year in advance of the first psychiatric hospitalization. Sleep disruption is a common experience for people with psychiatric disorders [40, 41], and many individuals with MD show circadian shifting [42], which results in a preference for being awake/active late at night. Precisely when sleep disturbances begin, however, is less well understood. According to our data, sleep dysfunction appears to already be present well in advance of the first psychiatric hospitalization and significant alterations in search timing occurred in both patient populations closer to the date of the first psychiatric hospitalization. As with search frequency, it remains unclear if different temporal patterns represent a change from baseline activity due to emerging psychiatric symptoms, or rather a persistent irregularity in sleep contributing to the later development of a psychiatric disorder. In either circumstance, extracting online search activity in youth presenting with sleep disturbance may one day serve as useful collateral information to predict the risk of psychiatric illness development.

Linguistic analysis of search terms identified significant differences in search content over 52-weeks before the first psychiatric hospitalization. Compared to HV, search terms among participants with SSD and MD demonstrated a greater emphasis on sadness, and perception, as well as first and second person pronouns. Several linguistic differences existed well in advance of the first psychiatric hospitalization and it is possible that certain linguistic features represent a state rather than a trait marker of mental illness. Analyzing word choice could therefore help to identify people at higher risk of SSD or MD prior to the emergence of clinically significant symptoms. As symptoms progressed, closer to the date of hospitalization, the content of searches changed significantly among individuals with SSD, but not MD. These linguistic changes may reflect shifting interests, changing mood, preoccupations, social functioning, and other domains known to accompany psychotic illness emergence [4]. In contrast to prior work exploring changes in language use on social media associated with relapse [29], participants with SSD were less likely to search for content related to perceptions, anger, and negative emotions. This may be related to differences in how people compose searches, which are private and generally intended to find information, versus social media posts, which are public and may be more likely to be communicating information.

Prior research in linguistic analysis has identified significant differences at the word level in the use of certain word categories, as well as at the sentence level in terms of semantic density, coherence, and/or content, both in individuals at risk for developing psychotic disorders as well as those with established SSD and MD [43–58]. Language extracted from online search activity is distinct due to its short sentence structure and the unique nature of the online search platform. As we continue to identify linguistic associations with psychiatric illness, the source of the language data must be taken into consideration. Future work is needed to better understand the clinical correlates of changing online search content and to identify the point in illness progression at which linguistic shifts emerge, in order to make the best clinical use of this information. Additionally, further analysis is needed to identify how language varies depending on the platform (Facebook vs Google, for example) used, and which has greater clinical utility.

Several noteworthy limitations should be mentioned. First, our sample size is relatively small and limits the generalizability of these findings. Second, the majority of our participants had medical record documentation that began with the first psychiatric hospitalization, making it challenging to know what symptoms were present and for how long prior to hospitalization. Given that many individuals report extended periods of untreated illness or comorbid psychiatric conditions prior to receiving clinical attention [5–8], it is possible that more data, beyond 52-weeks, is needed to identify shifts in search activity associated with illness progression. Additionally, future studies should consider monitoring participants prospectively and leveraging rating scales to more accurately explore how individual fluctuating interests and psychiatric symptoms impact search behaviors over time. Third, the fact that some participants searched more than others, may also impact results as there were large differences in the amount of extractable data across participants. While we do not anticipate that Google data alone will ever be sensitive or specific enough to establish a particular diagnosis, important questions for future research will be how much search data is necessary to make a reliable clinical prediction and how individual characteristics influence search behavior within a diagnostic group. Finally, eligibility criteria ranged from 15 to 35 years to reflect the inclusion criteria of the Early Treatment Program, however adolescents may engage with the Internet in a distinct manner compared to young adults and future initiatives will need to consider the impact of age as well as other demographic characteristics, such as sex, and education level on search activity.

Search patterns hold promise for gathering objective, non-invasive, and easily accessed, indicators of psychiatric symptom emergence. Utilizing online activity as collateral behavioral health information would represent a major advancement in efforts to capitalize on objective digital data to improve mental health screening. This would be a significant step forward for psychiatry, which has historically been limited by its reliance on self-reported data. However, how to effectively and ethically incorporate personalized patterns of online activity into public health initiatives and clinical workflow are critical questions [59]. The data utilized in the current study were obtained from consenting participants who were fully informed of the risks and benefits of participation. Furthermore, the data were extracted and analyzed locally at Northwell Health and remained entirely within a HIPAA compliant secure database to ensure the privacy of our participants. Nonetheless, this field of research evokes a host of challenging questions and concerns related to ethics, privacy, consent, and clinical responsibility. Interdisciplinary teams of researchers, clinicians, and patients must continue to work together on identifying and solving these important ethical dilemmas. Importantly, investigators must develop standards to protect the confidentiality and the rights of this sensitive population to avoid misuse of personal information and ensure that the data and the technologies are used in the service of positive outcomes for clinicians and the patients they treat.
